# Perivascular adipose tissue (PVAT) in atherosclerosis: a double-edged sword

**DOI:** 10.1186/s12933-018-0777-x

**Published:** 2018-10-10

**Authors:** Xiao-Yan Qi, Shun-Lin Qu, Wen-Hao Xiong, Oren Rom, Lin Chang, Zhi-Sheng Jiang

**Affiliations:** 1 Institute of Cardiovascular Disease, Key Lab for Arteriosclerology of Hunan Province, University of South China, Hengyang, 421001 China; 2 Cardiovascular Research Center, University of Michigan, Ann Arbor, MI USA

**Keywords:** Atherosclerosis, Perivascular adipose tissue, Adipokine, Inflammation

## Abstract

Perivascular adipose tissue (PVAT), the adipose tissue that surrounds most of the vasculature, has emerged as an active component of the blood vessel wall regulating vascular homeostasis and affecting the pathogenesis of atherosclerosis. Although PVAT characteristics resemble both brown and white adipose tissues, recent evidence suggests that PVAT develops from its own distinct precursors implying a closer link between PVAT and vascular system. Under physiological conditions, PVAT has potent anti-atherogenic properties mediated by its ability to secrete various biologically active factors that induce non-shivering thermogenesis and metabolize fatty acids. In contrast, under pathological conditions (mainly obesity), PVAT becomes dysfunctional, loses its thermogenic capacity and secretes pro-inflammatory adipokines that induce endothelial dysfunction and infiltration of inflammatory cells, promoting atherosclerosis development. Since PVAT plays crucial roles in regulating key steps of atherosclerosis development, it may constitute a novel therapeutic target for the prevention and treatment of atherosclerosis. Here, we review the current literature regarding the roles of PVAT in the pathogenesis of atherosclerosis.

## Background

Atherosclerosis is an inflammatory disease of the arteries characterized by lipid accumulation within the artery walls. In humans, atherosclerotic plaques are usually found in the aorta, the coronary arteries and cerebral arteries, but also in peripheral arteries [1]. Advanced atherosclerotic plaques grow large to block blood flow resulting in various cardiovascular diseases (CVD) including coronary heart disease (CHD), angina, carotid artery disease, peripheral artery disease and chronic kidney disease. Even though epidemiological and experimental studies have strengthened the pathophysiological associations between obesity and atherosclerosis [2, 3], the underlying causes of atherosclerosis remain unclear. Growing evidence suggest that atherosclerotic plaque formation begins with endothelial damage caused by factors released from adipose tissues to the circulation, promoting adhesions of circulating immune cells that initiate the progression of atherosclerosis [4, 5].

Adipose tissue is distributed throughout the body. In general, there are two main types of adipose tissues: white adipose tissue (WAT) and brown adipose tissue (BAT) [6, 7]. Historically, adipose tissue was recognized as a protective pad for neighboring organs. In recent years however, adipose tissue has emerged as a major endocrine organ which produces large amount of adipokines such as leptin, resistin, adiponectin and inflammatory cytokines [8–10]. Excess lipid accumulation in WAT causes adipocyte hypertrophy and dysfunction, resulting in enhanced secretion of deleterious adipokines and inflammatory cytokines into the circulation, that subsequently impair the function of the endothelium in blood vessels [11, 12]. Unlike WAT, BAT can take-up lipids to produce heat by uncoupling oxidation on mitochondrial electron transportation chain, which contributes to clearance of plasma lipids and prevents storage of lipids in WAT and other organs [13]. Dysfunctional WAT might be positively associated with atherosclerosis development, whereas activation of BAT may protect against atherosclerosis development. This hypothesis is strengthened by evidence of reduced hypercholesterolaemia and atherosclerosis development upon BAT activation with β3-adrenergic receptor stimulation in hyperlipidemic mice [14]. Key milestones in BAT research are the discovery of active BAT sites in adult humans and the “browning” ability of WAT by hormonal or cold temperature stimuli [15–18]. Browning of WAT is now defined as the third type of adipose tissue—beige adipose tissue (BeAT), which is characterized by high expression of the brown adipocyte marker uncoupling protein-1 (UCP-1) [19].

**Fig. 1:**
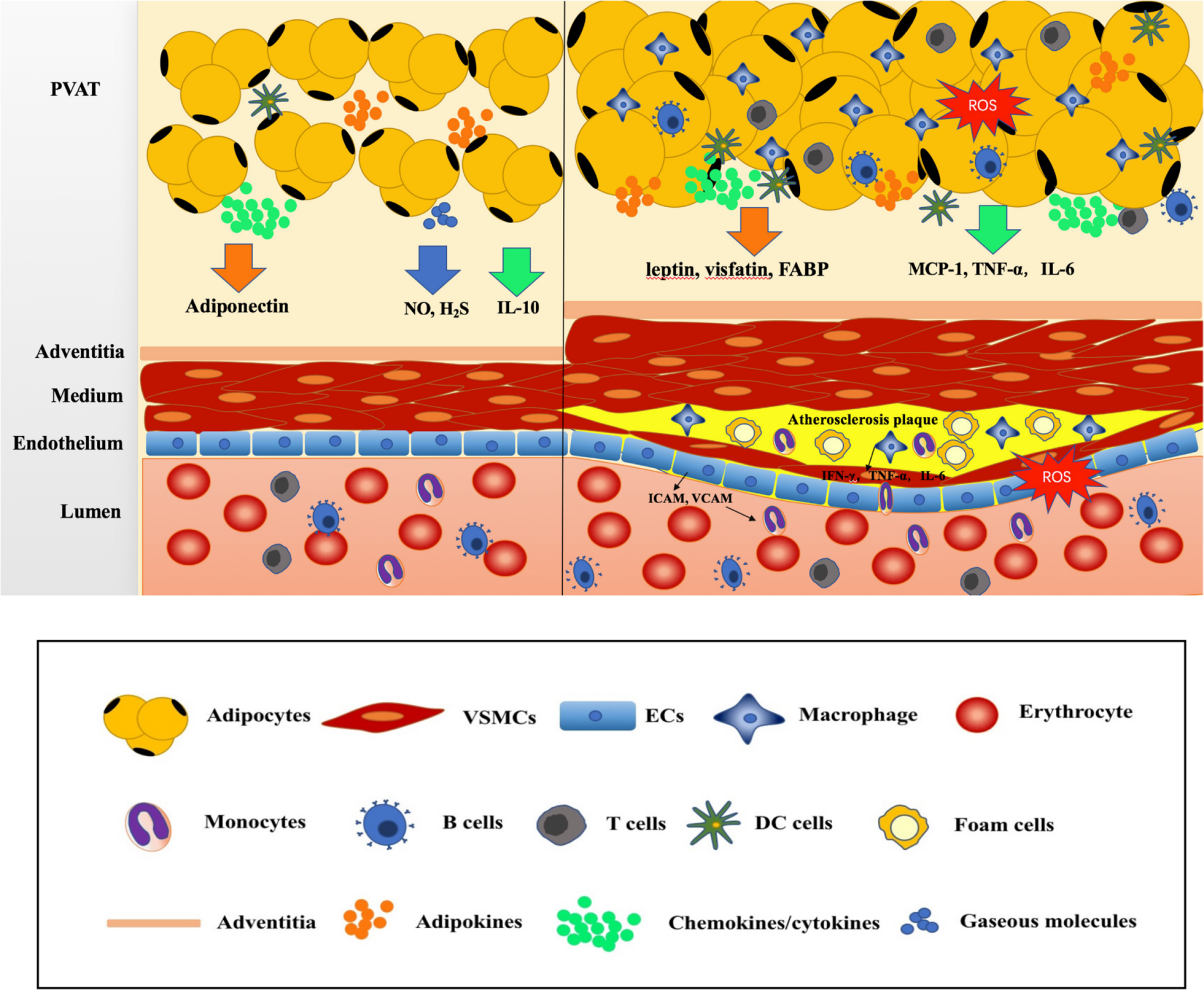
The role of PVAT in the regulation of atherosclerosis. In normal physiological state, PVAT secret protective adipokines and bioactive molecules such as adiponectin and NO maintain vascular homeostasis. Under pathology condition, unbalanced PVAT-derived adipokines, chemokines and cytokines targets many cell types including ECs, macrophage, T cells and SMCs involved in endothelial dysfunction, immune cells infiltration, smooth muscle cell migration and proliferation which are predominantly implicated in the pathological process of atherosclerosis

In addition to the classification of adipose tissues by their coloration, adipose tissues can also be categorized according to their anatomic locations. Perivascular adipose tissue (PVAT) is a type of adipose tissue which surrounds the blood vessels. Recently, PVAT characteristics resemble both BAT and WAT, it has been considered as an active component of the blood vessel walls, and involved in vascular homeostasis [20]. There are intensive studies regarding the relationship between PVAT and atherosclerosis [21, 22]. In this review, we will focus on the roles of PVAT in the pathophysiological progress of atherosclerosis (both pro- and anti-atherogenic effect) and its potential as a target for therapeutic intervention (Fig. 1).

## The characteristics of PVAT

Although surrounding adipose tissue is abundant in vascular trees of animals and humans, it was ignored for a long time. As pointed recently by Caroline Pond: “*human and comparative anatomists still regarded it as too inconsistent and inconsequential to be worthy of topographic, functional or evolutionary study. It was always dissected off vessels and lymph nodes in pickled prosections*” [23]. In 1982, Hausman and Richardson first described the histochemical and ultrastructural criteria of preadipocytes in perivascular location [24]. In 1984, de Souza et al. [25] described that the vessel’s sheaths and lamellae are formed by networks of collagen and elastic fibers. The perivascular area is filled by connective tissue, delimiting lobules of adipose tissue. Since then, there had been little research on the function of perivascular connective tissue until 1991 when Soltis and Cassis defined PVAT and its influence on rat aortic smooth muscle responsiveness [26]. In 2001, Okamoto et al. [27] reported the invasion of leukocytes into the perivascular adipose layer in response to angioplasty injury of coronary arteries, suggesting that PVAT inflammation might be associated with CVD.

In humans, the severity in spontaneous atherosclerosis may vary not only in different arteries but also in different segments of the same blood vessel [28]. Previous research, mainly in rodents, indicates that the appearance of PVAT varies by anatomic and metabolic context [29]. Current reports indicate that PVAT in the thoracic aorta is more similar to BAT, while PVAT in the abdominal aorta resembles both WAT-like and BAT-like phenotypes, which is referred as BeAT. Other smaller arteries are surrounded by WAT-like PVAT [30]. In humans, the coronary arteries are the most atherosclerosis-prone arteries with abundant of PVAT surrounding. In mice however, there is no PVAT surrounding the coronary arteries [30]. Of interest, mouse coronary arteries are resistant to atherosclerosis development [31], suggesting that the existence of PVAT may be associated with atherosclerosis development. However, the occurrence and development of atherosclerosis is influenced by many factors that determine the vascular susceptibility for atherosclerotic risk factors. Indeed, different segments of the aorta are differentiated from the distinct origins. It is unclear whether the atherosclerosis susceptibility of artery is associated with its origins. Similarly, different types of adipose tissue located at different anatomical depots may have their unique precursors.

The adipocytes in classical WAT such as subcutaneous WAT may be differentiated from mesenchymal stem cells (MSCs) in perivascular regions [32, 33]. Tang et al. [34] found that the majority of postnatal white adipocytes derived from prenatally peroxisome proliferator-activated receptor-γ (PPAR-γ) expressing lineage cells that are located in the stromal vasculature fraction (SVF). Gene-expression profiles showed that these PPAR-γ expressing lineage cells have a unique molecular signature and are phenotypically distinct from adipocytes and other stromal vasculature cells. Specifically, the SVF of harboring PPAR-γ expressing cells resembles blood vessels with expressions of mural cell markers smooth muscle actin (SMA), platelet-derived growth factor receptor, β polypeptide (PDGFRβ), and neural/glial antigen 2 (NG2). By using PDGFRβ or SM22Cre-mediated lineage studies, Tang et al. [34] further confirmed that PDGFRβ-expressing, but not SM22-expressing mural cells have adipogenic potential. Rodeheffer et al. [35] further identified that only CD29^hi^:CD34^+^ cells in SVF (about 59% cells in SVF) are of significant adipogenic differentiation populations. It is unknown whether mature brown adipocytes are differentiated from CD29^hi^:CD34^+^ precursors in SVF. Lee et al. [36] reported that white adipocytes and β3 agonist-induced brown adipocytes in abdominal WAT originated from the PDGFRα:CD34^+^Sca-1^+^ cells [36]. The same group further documented that cold stress rapidly induces de novo brown adipogenesis in classic BAT. Genetic lineage tracing demonstrated that newly generated brown adipocytes are derived from resident PDGFRα progenitors [37]. Additionally, interscapular brown adipocytes may share the same origin with skeletal muscle. Using in vivo fate mapping, Seale et al. [38, 39] reported that brown, but not white adipocytes arise from Myf5^+^ precursors, which was thought to be expressed only in the myogenic lineage. Even though PVAT shares many biochemical and morphological characteristics with classical brown or white adipocytes, it is unknown whether the adipocytes in PVAT are differentiated from precursors in WAT or BAT as described above.

Similar to classical brown adipocytes, beige adipocytes express UCP-1. However, brown and beige adipocytes may have different developmental origins [40]. Genetic deletion of PPAR-γ in vascular smooth muscle cells (VSMCs) using SM22a-Cre knock in tool, unexpectedly showed a phenotype that the adipose tissues in mesenteric region and peri-aortic region are completely undeveloped. However, adipose tissue in gonadal, subcutaneous, interscapular WAT and BAT were intact [41]. This mouse model strongly supports the notion that there are separate development origins for adipose tissues in different anatomic depots. Later, to address the cellular heterogeneity of different adipose tissue types, Long et al. [42] demonstrated the similarities and differences between brown and beige adipocytes in mice by applying translating ribosome affinity purification technology. Consistent with the report by Chang et al. [41], Long et al. [42] documented a smooth muscle-like gene expression signature (*Acta2*, *Tagln*, *Myh11*, *Myl9*, and *Cnn1*) in beige adipocytes, but not in interscapular classical brown adipocytes. By exposing Myh11-GFP/tdTomato mice to cold temperature for 2 weeks, which is known to induce browning in WAT, it was demonstrated that beige adipocytes arise from a Myh11^+^ precursor. Additionally, UCP1 positive lipid droplet-containing adipocytes were found in PRDM16-overexpressing mature VSMCs subjected to adipogenic cocktail and followed by treatment with triiodothyronine, rosiglitazone and insulin, indicating that mature VSMCs can give rise to adipocytes with a thermogenic gene-expression signature [42]. Whereas the above studies [41, 42] suggest that VSMCs might be the origin of some thermogenic adipocytes in PVAT or WAT, further studies are needed to clarify whether thermogenic adipocytes arise from SM22a^+^ or Myh11^+^ mature VSMCs or SMC-like stem cells. Therefore, these reports indicate that PVAT is distinct from the adipose tissues at other locations since the different origins.

As research focusing on PVAT characteristics was largely ignored until 2001, accumulating evidence suggests that PVAT is an active component of the blood vessel wall and contributes to vascular homeostasis by producing a number of biologically active molecules including adipokines (e.g. leptin, adiponectin, omentin, visfatin, resistin, apelin), cytokines/chemokines (e.g. interleukin-6, IL-6; tumor necrosis factor-α, TNF-α; monocyte chemoattractant protein-1, MCP-1), gaseous molecules (e.g. nitric oxide, NO, and hydrogen sulfide, H_2_S), prostacyclin, angiotensin-1 to 7 (Ang 1–7), Ang II, methyl palmitate, and reactive oxygen species (ROS) [20, 43–45]. Thus, PVAT-derived bioactive factors coordinately contribute to vascular tone regulations. PVAT-derived relaxing factors (PVRF) such as adiponectin, NO, H_2_S, prostacyclin, angiotensin1-7 and methyl palmitate can promote vasodilation by targeting the medial and the endothelial layer of blood vessels, whereas the PVAT-derived contractile factors (PVCF) such as Ang II, ROS and other unidentified factors may induce blood vessel constriction [46]. Therefore, PVAT is actively involved in the regulation of blood pressure. Circadian rhythm is one of the major physiological regulators of blood pressure, and is controlled by suprachiasmatic nucleus (SCN) where the circadian clock genes are coordinately expressed according to the light cycles. Recently, it was reported that circadian clock genes are expressed in PVAT which transcriptionally control local production of Ang II, suggesting that PVAT is involved in circadian regulation of blood pressure. Nevertheless, it remains unknown whether production of other PVRFs and PVCFs is also controlled in a circadian manner [47].

## PVAT and atherosclerosis

Atherosclerosis is a progressive chronic metabolic disorder which is characterized by endothelial dysfunction, lipid deposition and inflammation infiltration [48]. It is well-accepted that shear stress-induced endothelial dysfunction/damage is the initial step of the atherosclerosis development. Accordingly, atherosclerotic plaques are most frequently found at the bifurcation sites of the aorta/arteries [49]. Findings indicating that adhesions of circulatory inflammatory cells to the dysfunctional endothelium trigger cholesterol accumulation in the artery wall support the “inside to outside” theory of atherosclerosis development [50]. The atherosclerotic plaque is initiated and progressed in the neointima layer with intact internal elastic lamina, and the plaques are barely found in the media and adventitia layers [51]. However, the function of the endothelium of blood vessels is gradually deteriorating during the obesity process. Quesada et al. demonstrated that PVAT‐derived inflammatory mediators may adversely influence atherosclerotic plaque formation and stability [52]. Interestingly, PVAT also has been shown a protective role in endothelial dysfunction hypercholesterolemic LDL receptor deficient mice, suggesting that PVAT-derived factors might be involved in the regulation of endothelial function [53]. Thus, PVAT might actively influence atherosclerosis development in an “outside to inside” manner.

### Anti-atherogenic effects of PVAT

Atherogenesis is a closely associated with endothelial dysfunction that is characterized by reduced bioavailability of nitric oxide (NO). NO is produced by endothelial NO synthase (eNOS) which possess multiple anti-atherogenic properties including control of vascular smooth muscle proliferation and inhibition of platelet aggregation, leucocyte adhesion and vascular inflammation [54]. Recent studies show that eNOS is expressed not only in the endothelium but also in PVAT [55]. Moreover, PVAT-derived NO can be directly visualized in situ with fluorescence imagine [56]. In healthy individuals, basal NO production by small arteries is reduced when PVAT is removed, suggesting that PVAT contributes to vascular NO production [57]. Furthermore, PVAT can secret adiponectin which is known to normalize endothelial function by a mechanism that involves an increment in eNOS phosphorylation [58]. Another mechanism by which PVAT protects against atherosclerosis development involves the thermogenic ability result in clearance of plasma lipids from vasculature, another critical factor in atherosclerosis pathogenesis.

Previous evidence suggest different phenotypes of PVAT at different anatomical locations [59–61]. In the abdominal PVAT, white adipocytes are more abundant, whereas thoracic PVAT contains more brown adipocytes [61, 62]. Interestingly, microarray analysis revealed that the gene expression patterns in thoracic PVAT are almost identical to those of interscapular BAT in mice [61]. In contrast to BeAT, thoracic PVAT maintains a BAT-like phenotype under high fat diet feeding [61]. These findings suggest that thoracic PVAT more closely resembles classical BAT than BeAT in terms of morphology and function. Under physiological conditions, both WAT and BAT share anti-atherogenic properties. WAT acts as a lipid depot to prevent the rise lipids in the circulation, while BAT consumes large amounts of fatty acids via thermogenesis [63]. Indeed, activation of BAT protects against atherosclerosis development in mice [14]. Though the PVAT at different locations might show distinct morphology and function, it is accepted that PVAT inhibits atherosclerosis under physiological conditions via its thermogenic and fatty acid-scavenging capabilities. Recent studies have demonstrated that the cold exposure induce activation of thermogenesis in PVAT, accompanied by attenuation of the atherosclerotic process in apoE^−/−^ mice, whereas such protection is lost in mice where PVAT is absent [41], suggesting that the thermogenic properties of PVAT exert anti-atherogenic effects. In addition, PVAT-derived adipokines such as adiponectin, can suppress plaque formation and reduce inflammation indicating that the endocrine function of PVAT also have athero-protective effects [64]. Furthermore, growing evidence indicates that some endogenous bioactive gaseous molecules such as H_2_S, can alleviate atherosclerosis [65]. Although PVAT can secret such anti-atherogenic gaseous molecules, further investigation is needed to evaluate whether this might be another mechanism underlying the anti-atherogenic effects of PVAT. Taking together, the above evidence indicate that PVAT has anti-atherogenic properties involving improved endothelial function, metabolic and inflammatory responses.

### Pro-atherogenic effects of PVAT

Pediatric epidemiological studies documented that atherosclerosis begins in childhood [66]. The Bogalusa Heart Study showed that atherosclerotic lesions were found in young arteries aged 6–30 years [67]. The PESA (Progression of Early Subclinical Atherosclerosis) study indicated that the extent of subclinical atherosclerosis was present in 71% of men and 48% of women aged from 40 to 54 years [28]. These population studies strongly suggest that atherosclerotic lesions start at very young age. BAT exists in human infants, and is gradually replaced by WAT with growth. However, BAT is still present in adult humans and may be activated under specific condition such as cold stimuli [68]. PVAT resembles either WAT or BAT and emerged as a regulator involved in pathogenesis of vascular lesion formation.

Ketonen et al. reported that obesity-induced endothelial dysfunction in C57BL/6 mice is caused by enhanced expression of inflammatory cytokines and increased oxidative stress in PVAT [69]. In accord, Manka et al. found that transplantation of PVAT from obese mice to LDLR^−/−^ mice exacerbated lesion formation with increased inflammatory cell infiltration and pathological angiogenesis in adventitia [70]. Obesity is associated with increased risk for CVD and with remodeling of adipose tissues. It is clear that inflammation in adipose tissue under obese condition is highly related to atherosclerosis development [71]. Expansion of WAT and BAT due to obesity result in elevated basal lipolytic rate leading to enhanced release of fatty acids accompanied by secretion of pro-inflammatory factors that contribute to the development of insulin resistance (IR) and inflammation [11, 12, 72]. Additionally, IR leads to reduced clearance of fatty acids, which are deposited ectopically in muscle or liver, and enhances IR in turn [73]. Furthermore, fatty acids can activate Toll-like receptor (TLR) 2 and TLR4 which modulate the NF-κB signaling pathway in macrophages that play key roles in atherosclerosis development [74, 75]. Obesity-induced dysfunctional BAT undergoes a whitening switch, a phenotype which aggravates atherogenesis as evidenced by lipid accumulation and mitochondrial dysfunction [76, 77]. During obesity, PVAT, regardless of its phenotype or anatomical location, also becomes dysfunctional and releases elevated levels of pro-inflammatory factors, cytokines and chemokines directly to the vascular wall, inducing endothelial dysfunction and inflammation [78]. As obesity likely induces whitening of BAT-like PVAT, the thermogenic capacity of PVAT is reduced. Thus, obesity-induced PVAT dysfunction may cause endothelial dysfunction, immune cells infiltration, VSMCs migration and proliferation which contribute to atherosclerosis development. Since PVAT is adjacent to blood vessels, the paracrine effects of dysfunctional PVAT might have more pronounced consequences to atherosclerosis progression than adipose tissues in other depots.

## PVAT-derived adipokines and atherosclerosis

Adipocyte derived adipokines can modulate several physiological functions including food intake, glucose and lipid metabolism, thermogenesis, neuroendocrine function, blood pressure, and immunity [79]. Dysfunctional adipocytes under overweight/obesity conditions contribute to unbalanced release of adipokines which has been suggested as a potential link between PVAT and atherosclerosis. Atherosclerosis progression is tightly orchestrated by endothelial function, cholesterol transport, inflammation, immune response and VSMCs proliferation, all are intimately associated with adipokines (summarized in Table 1). Adiponectin is known to protect against atherosclerosis development. Adiponectin can suppress the generation of ROS [80], down-regulate the expression of adhesion molecules [81], and inhibit apoptosis [82]. Moreover, recent evidence emphasized that adiponectin is a negative modulator of innate immune response and can down-regulate the release of pro-inflammatory factors, TLR4 expression [83], promote cholesterol efflux from macrophage [84], and polarization of macrophages towards M2 phenotype [85]. Consistently, as an (AMP-activated protein kinase) AMPK agonist, adiponectin has anti-atherogenic properties [86–89]. In addition, vaspin, apelin and omentin-1 exert protective effects on atherosclerosis by inhibiting ROS generation, enhancing cholesterol efflux and reducing activation of inflammatory macrophages, respectively [90–92]. Unlike adiponectin, detrimental adipokines such as leptin, chemerin and resistin have been associated with endothelial cell proliferation, angiogenesis, ROS generation and adhesion molecules expression [93–95]. Particularly, leptin was shown to promote macrophage infiltration, VSMCs proliferation and release of TNF-α and IL-6 [96, 97]. Also, resistin facilitates macrophage recruitment in adipose tissue and atherosclerotic plaque [98], while leukocyte recruitment was shown to correlate with chemerin [99]. Clinical evidence indicating unbalanced adipokine profile (decreased serum adiponectin, apelin, omentin-1 levels and increased serum leptin, resistin, chemerin concentrations) in coronary atherosclerosis disease patients [100–105], further highlight the importance of adipokines as biomarkers for atherosclerosis.

**Table 1:** Role of adipokines in atherosclerosis

Adipocytokines	Role	Function	References
Adiponectin	Protective	Improve endothelial dysfunction Modulate immune response Promote cholesterol efflux Polarization of macrophages AMPK agonist	[80–82] [83] [84] [85] [86]
Vaspin	Protective	Inhibit ros generation	[90]
Apelin	Protective	Enhance cholesterol efflux	[91]
Ometin-1	Protective	Reduce activation of inflammatory macrophages	[92]
Leptin	Detrimental	Promote endothelial dysfunction Promote macrophage infiltration VSMCS proliferation	[93] [94] [97]
Resistin	Detrimental	Promote endothelial dysfunction Facilitate macrophage recruitment	[94] [98]
Chemerin	Detrimental	Promote endothelial dysfunction Promote leukocyte recruitment	[95] [99]
Visfatin	Detrimental	VSMCS proliferation Macrophage maturation Polarization of macrophages	[109] [114] [115]
FABP	Detrimental	Activate inflammatory macrophages	[116]
LCN-2	Detrimental	Activate inflammatory macrophages	[116]

Adipokines released by PVAT include adiponectin [106], leptin [107], resistin [108], visfatin [109], chemerin [110], lipocalin-2 (LCN2) [58], fatty acid binding protein (FABP) [111], which show direct evidence of PVAT-derived adipokines have effects on the progression of atherosclerosis. Accumulating evidence demonstrates a potential role of PVAT-derived adipokines in atherosclerosis. PVAT-derived adiponectin was shown to inhibit atherosclerosis by promoting macrophage autophagy via the Akt/FOXO3 signaling pathway [64] and to improve NO production by activating eNOS via PI3/Akt phosphorylation [112]. In contrast, PVAT-derived leptin promotes VSMC to undergo a switch into a synthetic phenotype via a p38 MAPK-dependent pathway which can be inhibited by leptin antagonist [107]. Leptin released by PVAT has also been shown to exacerbate coronary endothelial dysfunction through the protein kinase C-beta pathway [113]. Three newly discovered adipokines: visfatin, LCN-2, FABP represent a further link between adipose tissue and atherosclerosis due to their ability to activate macrophages and regulate their phenotypes [114–116]. Visfatin has been demonstrated to be secreted by PVAT and stimulate VSMCs proliferation in a dose- and time-dependent manner via extracellular signal-regulated kinase (ERK) 1/2 and p38 MAPK signaling pathways [109]. FABP4 locally produced by perivascular fat increased gene expression of inflammatory markers in a dose-dependent manner and was an independent predictor of the severity of coronary stenosis [111]. Nevertheless, further studies are warranted to show whether PVAT could secret all of those adipokines shown in Table 1 which have multiple functions during atherosclerosis process.

Taking together, under physiological condition, PVAT exerts anti-atherogenic effects partly through reducing inflammation, which are largely mediated by protective adipokines, such as adiponectin. However, in the setting of obesity in which inflammatory adipokines, such as leptin, are increased, the protective actions of PVAT are diminished. Thus, the balance between pro- and anti-inflammatory adipokines secreted from adipocytes determines the effects of PVAT on vascular remodeling processes.

## PVAT inflammation in atherosclerosis

Numerous reports support the key role of the inflammatory response in atherosclerosis development [117]. Leukocyte recruitment and pro-inflammatory cytokines participate pivotally in the early phase of atherogenesis. It is now well accepted that dysfunctional adipose tissue in conditions of obesity is a critical source of inflammatory factors that impact the cardiovascular system and promote atherosclerosis. In dysfunctional adipose tissue, secretion of adiponectin is reduced, whereas secretion of pro-inflammatory cytokines is increased, leading to enhanced local inflammation and substantially affecting cardiovascular function and morphology. Whereas earlier studies on inflammation in atherosclerosis have focused on neo-intima and atherosclerotic plaques, it is becoming clear that the inflammatory response in the pathogenesis of atherosclerosis not only occurs in the luminal side of the vessels, but also in the adventitial side [118]. A recent study demonstrated that PVAT and adventitial inflammation precede endothelial dysfunction and atherosclerotic plaque formation [119]. Interestingly, growing evidence suggests that macrophage infiltration is more pronounced in the adventitia than in the intima in animal models, making PVAT inflammation a critical player in atherosclerotic development [120, 121].

PVAT adipocytes and immune cells release vasoactive molecules such as adiponectin and IL-10 which have anti-inflammation effect under physiological conditions [83, 122]. Also, microarray analysis of perimesenteric adipocytes revealed increased expression of certain anti-inflammatory genes including tumor necrosis factor α-induced protein 6 (TAIP6) and suppressor of cytokine signaling 2 (SOCS2) [123]. On the other hand, transplantation of PVAT on the carotid artery resulted in increased vascular remodeling after wire-induced adventitial inflammation and angiogenesis in LDLR knockout animals [70]. Like other adipose depots, PVAT expands and become dysfunctional, then infiltrated with inflammation under pathological conditions [124–126]. Initiation of inflammation in PVAT results in enhanced secretion of chemokines and cytokines, such as MCP-1 which recruits cells, monocytes and macrophages. Immune cells derived IL-6 and TNF-α induce endothelium damage and increased expression of vascular cell adhesion molecule-1 (VCAM-1) and intercellular adhesion molecule-1 (ICAM-1), consequently triggering atherosclerosis development [127, 128]. Importantly, the direct contact of PVAT with the vessels enables significant anti- or pro-inflammatory signaling, mediated by immune cells, adipokines, chemokines and cytokines, that play key roles in atherosclerosis development.

### PVAT-derived chemokines and cytokines

Chemokines play crucial roles in initiation and regulation of inflammation, especially the immune response [129]. Chemokines are small molecules (8–12 kDa) which can be divided into 4 canonical subclasses: C, CC, CXC, and CX_3_C chemokines, based on the position of the N-terminal cysteine [130]. Chemokines and their receptors are widely expressed in vascular cells, such as endothelial cells, VSMCs and leukocytes [130]. In particular, the role of the CCL2–CCR2, CCL5–CCR1/CCR5 and CX_3_CL1–CX_3_CR1 chemokine axes has been well-established in atherogenesis [110, 131–135]. PVAT-derived chemokines induce recruitment of monocytes and T cells which in turn produce additional chemokines, resulting in enhanced infiltration of inflammatory cells in a positive feedback mechanism [136, 137]. Cytokines from dysfunctional PVAT induce VSMCs proliferation, endothelial dysfunction, enhanced secretion of pro-inflammatory adipokines and suppressed release of anti-inflammatory adipokines [123, 138, 139]. Most evidence points to the key role of IFN-γ, IL-17, IL-6 and TNF-α in regulating this pathogenic process, whereas the opposing action of IL-10 can improve endothelial function via inhibition of NADPH-dependent oxidative stress and increased NO production [140, 141]. The role of chemokines and cytokines in atherosclerosis is summarized in Table 2.

**Table 2:** Role of chemokines and cytokines in atherosclerosis

Mediators	Derived	Function	References
CCL2/ MCP-1	Adipocytes	Recruitment of T cells and monocytes Macrophage infiltration	[130, 286] [156]
CCL5/ RANTES	T cells, ECs VSMCs, adipocytes	Recruitment of T cells and monocytes	[130, 286]
CX3CL1	Monocytes ECs	Recruitment of T cells and monocytes Migration of VSMCs Anti-apoptosis and proliferative effects on monocytes and VSMCs	[130, 286] [287] [288, 289]
IFN-γ	Immune cells NK cells	Activation of monocytes/macrophages Polarization of immune cells Impair endothelium-dependent relaxation VSMCs proliferation and apoptosis	[290] [290] [137] [291]
IL-6	Macrophage T cells, ECs Adipocytes	Impair endothelium-dependent relaxation Superoxide production and endothelium dysfunction Th17 cell differentiation with pro-inflammatory effects on ECs and VSMCs VSMCs migration and proliferation	[292] [293, 294] [295] [296]
IL-17	Th cells	Expression of adhesion molecules in ECs and VSMCs Decreased NO production Collage deposition	[297] [298] [299]
TNF-α	Immune cells Adipocytes	Decrease eNOS expression, enhance ROS production Increase endothelial adhesion molecules and chemokines	[143, 300] [300]
IL-10	Treg cells Macrophage DCs	Reduce oxidative stress Increase production of NO Inhibit activation of P38 MAPK	[141] [141] [122]

### Immune cells in PVAT

#### T cells

Immune cell infiltration is a key driver of PAVT inflammation. In contrast to visceral fat, PVAT T cell infiltration may precede and exceed macrophage infiltration in animal models [142]. Perivascular T cells represent a morphologically and functionally heterogeneous cellular population. Both CD4^+^ T helper (Th), CD8^+^ T cytotoxic (Tc), T regulatory (Treg) and γ/δ T cells are present in PVAT [132, 143, 144]. Th1, Tc1 and Th17 cells are pro-atherogenic cells that secret pro-inflammatory cytokines, such as IFN-γ, TNF-α, IL-17 [145]. In contrast, Treg cells are atheroprotective cells that release anti-inflammatory cytokines (IL-10) which play a critical role in immune homeostasis and prevent excessive immune responses [144, 146]. Interestingly, a subset of CD8^+^ Treg cells control immune responses by affecting apoptosis and the function of adjacent vascular cells [147]. While other subpopulations of T cells such as invariant natural killer T (iNKT) cells have been reported in PVAT, which mainly contribute to production of IFN-γ and TNF-α [148]. Finally, γ/δ T cells have been demonstrated to represent a substantial proportion of T cells in PVAT, but their functional importance is not clear [149].

#### B cells

During atherosclerosis, B cells are primarily localized within the plaque and in artery tertiary lymphoid organs (ATLOs) [150]. The effects of B cell on atherosclerosis development are dependent on their subset, with B-1 cells attenuating and B-2 cells aggravating atherosclerosis. B-1 cells exert anti-atherogenic activities via secretion of IgM, inhibiting the formation of foam cells, whereas B-2 cells stimulate Th1 cells and dendritic cells to play a pro-atherogenic role [151]. Little is known about the characteristics of B cells and their function in PVAT, but a recent study provides evidences that the majority of B cells in and around the aorta are derived from PVAT in mice [152]. In addition, a large proportion of these B cells belong to the B-1 subset which secret significantly greater numbers of IgM secreting cells than the aorta. Moreover, ApoE^−/−^ mice with B cell-specific knockout of the gene encoding the helix-loop-helix factor Id3, which is known to attenuate diet-induced atherosclerosis, have increased numbers of B-1 cells and increased IgM secreting cells in PVAT. Most important, immunostaining of PVAT on human coronary arteries identified fat associated lymphoid clusters (FALCs) harboring high numbers of B cells, and flow cytometry demonstrated the presence of T cells and B cells including B-1 cells [152]. Taken together, these finds demonstrated that PVAT harbors atheroprotective IgM-producing B-1 cells.

#### Macrophages

Resident adipose tissue macrophages present antigens to lymphocytes, phagocytize pathogens, release antimicrobial peptides, and attract other immune cells [153]. Macrophages constitute about 10–15% of stromal-vascular fraction, while a dramatic increase of up to 45–50% is observed during obesity [154]. In hypercholesterolaemia and hypertension, macrophages can accumulate in PVAT and release free radicals via NADPH oxidase 2 (NOX2) even in the absence of obesity [155–157]. Under physiological conditions, M2 macrophages which produce IL-10 are dispersed in adipose tissues [158], while in pathological conditions, polarized M1 macrophages accumulate in crown-like structures surrounding adipocytes and produce pro-inflammatory cytokines such as IL-6, IFN-γ and TNF-α [159, 160]. In PVAT, macrophages play an important role in regulating T cell activation through antigen presentation and expression of co-stimulatory ligands [161]. Indeed, T cell-dependent responses may reciprocally regulate PVAT macrophage infiltration [162], indicating a bidirectional relationship between the T cells chemotaxis and macrophage infiltration in PVAT inflammation.

#### Dendritic cells (DC) and natural killer (NK) cells

Dendritic cells (DC) are the most efficient antigen presenting cells, which are located primarily on the adventitia–PVAT border, but have also been reported within PVAT [132, 163]. A close relationship between DC and T cells has been demonstrated. During PVAT inflammation, DC release inflammatory mediators (e.g. IL-1β, IL-6 and IL-23), promoting T cells to produce pro-inflammatory cytokines such as IL-17, TNF-α and IFN-γ [123, 164]. Moreover, blocking the CD28/CD80/CD86 co-stimulation axis between DC and T cells prevents PVAT inflammation [165]. Compared to DC, the role of NK cells in PVAT inflammation is less clear. As NK cells regulate macrophage polarization and insulin resistance through release of IFN-γ in visceral adipose tissue [166], further research focusing on the role of NK cells in PVAT is warrant.

## Cellular and molecular contact between PVAT and the vessel wall in the progression of atherosclerosis

Pathological studies have revealed a defined series of changes in the vessel wall during atherogenesis including endothelial dysfunction, inflammatory cells infiltration, foam cell formation, lipid overload, SMCs migration and proliferation and fibrous plaque formation. PVAT releases biologically active molecules and secretes adiopokines/cytokines that can modulate pathophysiological process of atherosclerosis [20]. The possible role of PVAT in the initiation and progression of atherosclerosis is detailed below.

### Lesion initiation—Endothelial dysfunction

The endothelium functions as a selective permeable barrier between blood and surrounding tissues. The endothelium also produces effector molecules that regulate key processes such as, inflammation, vascular tone, vascular remodeling and thrombosis [167]. It is well-accepted that endothelial dysfunction is a hallmark feature of the initiation of atherosclerosis [168]. Damaged endothelium promotes atherosclerosis by increasing leukocyte adhesion, vascular permeability to lipoproteins, platelet aggregation and generation of cytokines [169]. As mentioned earlier, PVAT releases a number of protective agents under physiological conditions, such as NO, H_2_S and adiponectin. These are well-known as protective endothelium-derived factors with anti-atherogenic properties. Indeed, the lack of NO and H_2_S accelerates the progression of atherosclerosis [170, 171]. During obesity, PVAT-derived NO, H_2_S and adiponectin are decreased in obese animals [172–174], and the hypertrophy of adipocytes in PVAT promotes endothelial dysfunction in relation to increased NADPH oxidase-derived oxidative stress and enhanced inflammation [69]. But in a human research, their data suggest increased adiponectin expression in perivascular tissue which might a compensatory mechanism to preserve endothelial function in obese patients [175]. For this point, there are some weaknesses of this study such as sample size, more rigorous investigations should be addressed.

### Lesion development—Macrophage recruitment and activation, foam cell formation and plaque rupture

The impact of PVAT on experimental atherosclerotic lesion development has been less extensively examined. Under homeostatic conditions, the anti-inflammatory effects of PVAT predominate, and secretion of pro-inflammatory paracrine agents is relatively low [176]. In one of the few studies available so far it was shown that PVAT-derived adiponectin was shown to suppress perivascular collar-induced carotid atherosclerotic lesion formation in high fat diet-fed apoE^−/−^ mice through increasing macrophage autophagy [64]. Macrophages accumulate in the vascular wall and take up modified lipoproteins (mainly oxidized-LDL) to form foam cells, a hallmark feature of early atherogenesis. This modification presumably involves ROS, secretory phospholipases A2 (sPLA2) produced by ECs and macrophage [177, 178]. The rapid uptake of highly modified LDL particles by macrophages is related with ‘scavenger’ receptors CD36 which is regulated by PPAR-γ [179, 180]. As clarified above, dysfunctional PVAT produces inflammatory adipokines/cytokines such as leptin, TNF-α and IL-6, which induce endothelium production of VCAM-1, ICAM-1 and MCP-1 promoting the adherence and migration of monocytes into the subendothelial layer of the intima [127, 128, 181, 182]. Once inside the intima, monocytes acquire characteristics of resident macrophages and secret pro-inflammatory cytokines such as IL-6, IFN-γ and TNF-α, aggravating recruitment of inflammatory cells, endothelial injury and LDL oxidation, then, accumulation of the modified LDL particles in these macrophages eventually turns into the foam cells [183]. On the other hand, our previous data showed that selective depletion of PVAT in mice with PPAR-γ deletion in SMCs was associated with impaired vascular thermoregulation, endothelial dysfunction [41]. Morover, we further demonstrate that brown adipocyte-specific PPAR-γ deletion impairs PVAT development and enhances atherosclerosis in mice suggesting that PPAR-γ could be a pivotal link between PVAT and atherosclerosis lesion development [184]. In more advanced stages of atherosclerosis, macrophages induce apoptosis of VSMCs that requires a combination of at least Fas Ligand and NO [185, 186]. Finally, activated macrophages express effector molecules such as matrix mentalloproteinases that promote rupture of atherosclerotic plaques [187, 188]. Taken together, increased expression of inflammatory adipokines/cytokines in dysfunctional PVAT enhances endothelial dysfunction, leading to macrophage recruitment and activation that promote vascular lesion formation and progression.

### Neointima formation—Smooth muscle cells proliferation and migration

Neointima are characterized by accumulated VSMC and VSMC-derived extracellular matrix which contribute to further stenosis. The effects of PVAT on intimal SMC infiltration have been investigated after transplantation of thoracic PVAT to wire-injured carotid arteries, where the presence of PVAT accelerated neointimal formation in an MCP-1 dependent manner [70]. Also, transplantation of PVAT derived from transgenic mice overexpressing adipose tissue-specific angiopoietin like 2 (ANGPTL2) accelerated neointimal hyperplasia induced by wire injury of the femoral artery, which was attenuated by transplantation of PVAT from ANGPTL2^−/−^ mice [189]. Moreover, inflamed PVAT increases VSMC proliferation in a TGF-β dependent manner, suggesting that TGF-β released from PVAT can potentiate neointima formation [190]. Furthermore, as reviewed elsewhere [136], PVAT-derived factors such as leptin, viafastin, TNF-α, IL-6 and IL-8, promote VSMC proliferation and migration. However, it is noteworthy, that the role of VSMC migration and proliferation in atherosclerosis remains controversial, and proliferation of VSMC is mainly considered as a stabilizer of atherosclerotic plaques rather than contributing to their formation [191].

## PVAT: link between atherosclerosis and diabetes

It is worth mentioning that atherosclerosis is closely associated with and accelerated by metabolic syndrome, especially diabetes. Because the metabolic syndrome occurs in most people with type 2 diabetes, its presence likely accounts for most of the increased incidence of CVD in type 2 diabetes [192]. Furthermore, the presence of diabetes increases the risk of CVD beyond that with metabolic syndrome alone distinguishes it from metabolic syndrome [192]. Oxidative stress, inflammation and IR are key players in the development of atherosclerosis, diabetes and their complications. IR is a major feature of type 2 diabetes and develops in multiple organs, including skeletal muscle, liver, adipose tissue, and heart. Clinical hyperglycemia is always preceded by many years of IR which is associated with obesity [193]. Indeed, a substantial proportion of diabetic patients are obese [194]. Adipose tissue is main sources of free fatty acid (FFA) and pro-inflammatory molecules [195]. Hypertrophy of adipose tissue release abundant FFA binding TLRs to phosphorylation of insulin receptor substrate-1 (IRS-1), result in the down-regulation of the glucose transporter-4 (GLUT-4) and, hence, IR [196, 197]. Phosphorylated IRS-1 alters its ability to activate downstream PI3-kinase and Akt leads to eNOS inhibition and decreased NO production [196]. Furthermore, intracellular oxidation of stored FFA generates ROS leading to vascular inflammation, advanced glycation end products (AGEs) synthesis and protein kinase C (PKC) activation [198, 199]. The initial trigger vascular function in diabetes is the hyperglycemia related reduced NO bioavailability and accumulation of ROS, leading to endothelial dysfunction [200]. Actually, overproduction of ROS by mitochondria is the causal link between high glucose concentration and biochemical pathways of vascular complications in diabetes. Indeed, hyperglycemia-induced ROS production triggers several cellular mechanisms including AGEs sythesis, PKC activation, and NF-κB-mediated inflammation [199, 201]. PKC and its downstream targets play a major role in vascular dysfunction. PKC not only trigger eNOS upcoupling [202–204] but also increase synthesis of endothelin-1 favouring vasoconstriction and platelet aggregation [205]. Moreover, accumulation of superoxide anion also triggers up-regulation of pro-inflammatory genes MCP-1, VCAM-1, and ICAM-1 via activation of NF-κB signaling [199]. More interesting, glucose intolerance is related with down-regulated PPAR-γ in adipose tissue which impairs development of PVAT and enhances atherosclerosis [184, 206]. Nonetheless, hyperglycemia and IR alone may not fully explain the persistent cardiovascular risk burden associated with type 2 diabetes. Exactly, normalization of glycemia does not reduce macrovascular events suggesting that mediators of other risk factors other than glucose significantly participate to increase the residual cardiovascular risk in diabetic patients [207]. In this regard, adipose dysfunction, result in aberrant released adipokines/cytokines, oxidative stress, hypoxia and inflammation may be particular relevant [208]. One study has demonstrated that fed rat with fructose can induce the changes in PVAT fatty acyl composition and oxidative stress biomarkers which are associated with vascular reactivity. The PVAT redox state was also modified by the fructose overload, as shown by a reduced activity of antioxidant enzymes and augmented oxidative stress [209]. It has been proposed that dysfunction of PVAT is associated with reduced glucose transport by reducing muscle perfusion [210].

As clarified above, dysfunctional adipose leads to an altered secretory profile. Adipokines and cytokines are link to IR, oxidative stress, inflammation or immune response [211–213]. Adiponectin released from PVAT has been reported to affect insulin sensitivity, inflammatory responses, appetite, atherosclerosis [214]. In adiponectin knockout mice, adiponectin deficiency reduced IRS-1-mediated insulin signaling, resulting in severe diet-induced IR [215]. Furthermore, in obese animals, administration of adiponectin has been found to improve insulin sensitivity and to decrease hyperglycemia and the level of fatty acids in the plasma [216, 217]. Enlargement of adipose tissue is accompanied by reduced expression of adiponectin and increased release of pro-inflammatory cytokines such as TNF-α and IL-6 [214]. Whereas, another study has showed a causal relationship between high serum adiponectin levels and increased cardiovascular mortality rate in patients with type 2 diabetes that pointing to an unexpected deleterious action [218]. The intrinsic biological nature underlying deleterious effect is difficult to understand or only to speculate. Whether adiponectin play a role in increasing atherosclerotic processes is a possibility that deserves further, specifically designed studies. Conversely, in another study, leptin expression was increased in the PVAT, leading to inflammation, fibrosis and vascularization, in patients undergoing coronary artery bypass surgery [219]. Moreover, there is a correlation between increases in plasma leptin and IR and cardiac dysfunction in a type 2 diabetes model [220]. The effects of leptin leading to IR may be indirectly mediated by the brain where it activates the sympathetic nervous system [221]. Another adipokine, resistin, has been described to increase TNF-α and IL-6 production [222]. In endothelial cells, resistin has been found to increase VCAM-1 and ICAM-1 expression [223]. Irisin, a new hormone released by adipose tissue may be involved in pro-atherogenic endothelial disturbances that accompany obesity and T2DM [224]. Omentin-1, a novel adipocytokine mainly expressed in visceral adipose tissue, has been found to inhibit the inflammatory response and improve insulin resistance as well as other obesity-related disorders. Recently, circulating and epicardial adipose tissue (EAT)-derived omentin-1 levels were reduced in patients with CAD. Furthermore, omentin-1 expression in patients with CAD was lower in EAT adjacent to coronary stenotic segments than non-stenotic segments. Whether EAT omentin-1 expression associated with local coronary atherosclerosis should be further clarified [225]. Inflammatory cytokines such as TNF-α may be associated with obesity-related IR. In TNF-α deficient obese mice had lower levels of circulating FFA and were protected from the obesity-related reduction in the insulin receptor signaling in muscle and fat tissues [226]. These findings demonstrated that TNF-α is an important mediator of IR in obesity through its effects on several important sites of insulin action. Taken together, adipose tissue is a resource of multiple active molecules that influence the common pathogenesis of atherosclerosis and diabetes. PVAT attracts more attention as its unique location but more direct evidence should be shown to demonstrate its pivotal function in metabolic disease and cardiovascular system.

## Therapeutic targeting on PVAT

### PPAR-γ agonist and the adiponectin axis

PPAR-γ is an important regulator of adipocyte function, with diverse effects on whole body glucose and lipid metabolism. Activation of PPAR-γ in a variety of tissues such as the liver and skeletal muscle ameliorates insulin resistance [227]. Evidence have demonstrated that loss of PVAT on PPAR-γ deletion in smooth muscle cells or in BAT impairs intravascular thermoregulation and enhances atherosclerosis, indicating PPAR-γ as a key mediator between PVAT and atherosclerosis [41, 184]. Thiazolidinediones (TZD), including rosiglitazone and pioglitazone, are a class of anti-diabetic drugs that were reported to possess also anti-atherogenic and anti-inflammatory effects [228]. More important, pioglitazone may offer a new effect on the whole vessel wall, promoting the stability of advanced atherosclerotic plaques [52]. However, it should be noted that TZD use is associated with the risk of fluid retention which may exacerbate heart failure [229]. This highlights the need for better understanding of the tissue-specific effects of PPAR-γ, in order to target its signaling more effectively.

To date, adiponectin is one of the most studied adipokines which is a known downstream target of PPAR-γ [230, 231]. A recent study group use TZD to stimulate adipogenesis in precursor cells emerging from from thoracic and abdominal aortic rings. They observed that cells emerging from the aortic ring take on adipocyte morphology, express adipocyte markers, and secrete adiponectin suggesting TZD can not only promote perivascular adipogenesis but also induce secretion by PVAT [232]. Numerous reports have identified a plethora of anti-inflammatory, insulin-sensitizing, and anti-oxidative roles for adiponectin. Indeed, PVAT-derived adiponectin directly related to endothelial function [58], where it suppresses the generation of ROS [80], and down-regulates adhesion molecules expression [81]. In addition, adiponectin inhibits atherosclerosis by aggravating macrophage autophagy [64]. Additionally, adiponectin induces M2 macrophage polarization while inhibiting inflammatory infiltration and reducing lipid content in adipose tissue [233]. Pharmaceutic adiponectin now still targeting its receptor and an oral adiponectin receptor agonist have been demonstrated to improve insulin sensitivity and glucose tolerance in mice [234], suggesting that signaling of adiponectin may be beneficial in vivo, but its significance in vascular disease remains to be evaluated.

### GLP-1 receptor agonists/analogues and DPP-4 inhibitors

Glucagon-like peptide-1 (GLP-1) receptor agonists/analogues or dipeptidyl peptidase-4 (DPP-4) inhibitors are anti-diabetic drugs also shown to have beneficial effects against vascular disease [235, 236]. Interestingly, adipose tissue also secretes low levels of GLP-1, which reduces lipid accumulation [237], increases the expression of adiponectin [238], and promotes M2 macrophage polarization [239]. The specific effects of these agents on PVAT and whether they can be involved in paracrine cross-talk between PVAT and the vascular wall are still questioned. DPP-4 enzymatically modifies GLP-1, facilitating its degradation and reducing its bioavailability. It is reasonable to assume that the effects of DPP4 inhibitors on AT and CVD will derive from the concomitant potentiation of GLP-1 actions. But recent evidence indicate that the effect of DPP-4 on adipose tissue and vasculature are beyond GLP-1. DPP-4 that is able to modulate of endothelial progenitor cells, inflammatory pathway and ischemic response [240]. Hence, DPP-4 inhibitors exert its anti-atherogenic effect through not only GLP-1 dependent way but also independent way. For specific target on PVAT, teneligliptin, a DPP-4 inhibitor attenuated atherogenesis with alter the inflammatory phenotype in PVAT [241, 242]. Therefore, targeting of DPP4 may be a crucial regulator of the cross-talk between PVAT and the vascular wall.

### Browning of WAT

An alternative therapeutic approach is to induce the formation of beige adipocytes in PVAT. Similar to classic brown adipocytes, beige adipocytes may also use triglycerides for non-shivering thermogenesis [243]. Thus, browning of PVAT will likely contribute to lowering of plasma triglyceride levels and preventing atherosclerosis. Cold temperature and catecholamine stimulation are well-established approaches to induce browning of WAT [14, 244, 245]. In addition, numerous approaches/stimuli that promote a brown-like phenotype are emerging, including activation of β3-adrenergic receptors [246], AMPK activation [247], inhibitors of Notch signaling [248], lactate [249], gut microbiota [250], thyroid hormone [18], fibroblast growth factor 21 [251], bone morphogenetic protein (BMP) 4 [252, 253] and BMP7 [253]. Furthermore, various components of the immune system including macrophages [254], eosinophils [255] and group 2 innate lymphoid cells (ILC2) [256] have been implicated to promote the browning process.

### Anti-inflammatory strategies

As described above, release of adipokines, chemokines and cytokines in dysfunctional PVAT potentially facilitates PVAT local inflammation and enhances atherosclerosis progression. To date, there are no effective strategies that directly target PVAT local inflammation. Increasing anti-inflammatory adipokines, decreasing inflammatory cytokines or suppressing immune cells infiltration seems to be a promising approach to reduce inflammation in PVAT. The existing anti-inflammatory drugs, anti-TNF monoclonal antibodies, exert mainly systemic vascular effects partially via elevation of circulating adiponectin levels [257]. Interestingly, PPAR-α agonist and statins, which are frequently used in the treatment of cardiovascular disease, have been shown to decrease adipose tissue inflammation by enhancing the action of adiponectin and inhibiting macrophage activation via the TLR4 signaling pathway [258–260]. Also, salicylates can promote macrophage polarization into anti-inflammatory (CD206^+^) M2 phenotype in WAT [261], and stabilize pre-existing atherosclerosis partly through inhibition of the NF-κB pathway [262]. In addition, dietary flavonoids and oestrogen have been suggested to alleviate inflammation in adipose tissue [263–265]. Whether the above approaches have direct effects on PVAT inflammation are remain unknown, and further investigations are needed to establish novel strategies to target local inflammation within PVAT.

### Inhibition of renin–angiotensin–aldosterone system

The renin–angiotensin–aldosterone system (RAAS) is involved in systemic blood pressure regulation and in renal electrolyte homeostasis. Except for renin, all of the RAS system components are expressed in adipose tissue [59]. Indeed, evidence has demonstrated the presence of local RAAS activity in PVAT [266]. AngII is the major component of RAAS with a variety of physiological actions including vasoconstriction, stimulation of aldosterone release from the adrenal gland, renal sodium and water reabsorption, increase of blood pressure, cell growth, inflammation process, vascular and cardiac remodeling, activation of the sympathetic nervous system, and ROS production [267]. Ang II exert its function through two main membrane receptors: the angiotensin type 1 receptor (AT1R) and the angiotensin type 2 receptor (AT2R). The AT1R is responsible for most biological effects of Ang II such as pressure, trophic, and pro-inflammatory effects. Sakaue et al. reported that ATR1 receptor in PVAT promotes vascular inflammation and aneurysm formation [268]. Previous research has reported angiotensin receptor blockers (ARBs) and angiotensin converting enzyme inhibitors (ACEI) reduced PVAT inflammation, which is associated with the reduction of atherogenesis [269]. Furthermore, some ARBs are also able to act as partial PPAR-γ agonists, up-regulating adiponectin expression [270]. In addition to Ang II, aldosterone is also an important mediator of RAAS effects which can inhibit physiological insulin signaling and promote local inflammation via activation of T cells and macrophages [271]. Recent work verifies the direct action of aldosterone on adipose tissue was associated with metabolic syndrome, insulin resistance and a pro-inflammatory phenotype of the adipose tissue utilizing an adipose tissue-specific mineralocorticoid receptor-overexpressing mouse model [272]. Mineralocorticoid receptor antagonists have revealed beneficial effects in terms of vascular redox state, systemic insulin sensitivity, vascular remodeling, endothelial function and AT inflammation [273–275]. Consequently, RAAS inhibitors such as ACEI, ARBs and aldosterone inhibitors may target adipose tissue to perform beneficial metabolic and cardiovascular effects.

### AMPK activators

AMPK is a serine/threonine protein kinase involved in the regulation of metabolism, such as glucose transport, mitochondrial function, fatty acid oxidation and inflammation [276]. AMPK is essential for the maintenance of cardiovascular health and several drugs commonly used for the treatment of CVD and metabolic diseases may also work through AMPK (e.g. statins, metformin and the TZD [277, 278]. Several studies revealed an irrefutable relationship between AMPK, obesity and inflammation [279, 280]. Activation of AMPK is known to exert an anti-inflammatory response via up-regulating IL-10 and down-regulation of TNF-α and IL-6. Abnormal lipid profiles and lipotoxicity, as seen in obesity and T2DM, predispose individuals to CVD. AMPK is a major regulator of lipid metabolism via stimulation of lipid oxidation [281, 282]. Indeed, it was reported that PVAT induces vascular dysfunction via dysregulation of the AMPK/mammalian target of rapamycin (mTOR) pathway in high fat diet-induced obese rats [283]. Treatment of PVAT from rat thoracic aorta with AMPK activators AICAR, salicylate, metformin, resveratrol or diosgenin down-regulates the expression of proinflammatory cytokines (TNF-α, IL-6, MCP-1) and increases anti-inflammatory factors (adiponectin, PPAR-γ) in PVAT, which is associated with an increased eNOS phosphorylation and improved PVAT function [284, 285]. Thus, targeting AMPK in PVAT may have an anti-atherogenic therapeutic potential.

## Conclusion

PVAT plays key tissue-specific roles during the development of atherosclerosis. Under physiological conditions, PVAT is able to store and combust lipids, generate heat and take up fatty acids from the blood. PVAT also releases multiple vasoactive molecules such as NO, H_2_S and adiponectin, which protect against atherosclerosis development. Under pathophysiological conditions (e.g. obesity, hyperlipidemia and diabetes), PVAT becomes dysfunctional. Together with reduced thermogenic capacity due to whitening of BAT-like PVAT, dysfunctional PVAT releases pro-inflammatory adipokines that promote endothelial dysfunction, infiltration of inflammatory cells and migration of VSMC, subsequently promoting atherosclerosis development. Thus, targeting PVAT function is emerging as a novel therapeutic approach for the treatment of atherosclerosis that warrants validation in future research.

In summary, accumulating evidence indicate PVAT as a double-edged sword that possess both anti-atherogenic and pro-atherogenic effects under different conditions. Further study is needed to evaluate whether targeting PVAT function can be used as a novel approach for the treatment of atherosclerosis and subsequent CVD.

## Abbreviations

Ang 1-7: angiotensin-1 to 7
AT1R: angiotensin type 1 receptor
AT2R: angiotensin type 2 receptor
ARBs: angiotensin receptor blockers
ACEI: angiotensin converting enzyme inhibitors
AGEs: advanced glycation end products
BAT: brown adipose tissue
BeAT: beige adipose tissue
BMP: bone morphogenetic protein
CVD: cardiovascular diseases
DCs: dendritic cells
eNOS: endothelial nitric oxide synthase
FABP: fatty acid binding protein
FALCs: fat associated lymphoid clusters
FFA: free fatty acid
H_2_S: hydrogen sulfide
GLUT-4: glucose transporter-4
IR: insulin resistance
ICAM-1: intercellular adhesion molecule-1
IL: interleukin
LCN2: lipocalin-2
IRS-1: insulin receptor substrate-1
MSCs: mesenchymal stem cells
MMPs: matric mentalloproteinases
MCP-1: monocyte chemoattractant protein-1
NO: nitric oxide
NG2: neural/glial antigen 2
PVRF: PVAT-derived relaxing factors
PVCF: PVAT-derived contractile factors
PDGFR: platelet-derived growth factor receptor
PKC: protein kinase C
ROS: reactive oxygen species
RAAS: renin–angiotensin–aldosterone system
SVF: stromal vasculature fraction
SMA: smooth muscle actin
SCN: suprachiasmatic nucleus
TLR: Toll-like receptor
TAIP6: tumor necrosis factor α-induced protein 6
TNF-α: tumor necrosis factor-α
UCP-1: uncoupling protein-1
VSMCs: vascular smooth muscle cells
VCAM-1: vascular cell adhesion molecule-1
WAT: white adipose tissue
